# Energy-Efficient Hosting Rich Content from Mobile Platforms with Relative Proximity Sensing

**DOI:** 10.3390/s17081828

**Published:** 2017-08-08

**Authors:** Ki-Woong Park, Younho Lee, Sung Hoon Baek

**Affiliations:** 1 Department of Computer and Information Security, Sejong University, Seoul 05006, Korea; woongbak@sejong.ac.kr; 2 Department of Industrial and Systems Engineering, SeoulTech, Seoul 01811, Korea; younholee@seoultech.ac.kr; 3 Department of Computer System Engineering, Jungwon University, Chungbuk 28024, Korea

**Keywords:** energy-efficiency, mobile communication, proximity sensing, mobile platform, content hosting

## Abstract

In this paper, we present a tiny networked mobile platform, termed Tiny-Web-Thing (*T-Wing*), which allows the sharing of data-intensive content among objects in cyber physical systems. The object includes mobile platforms like a smartphone, and Internet of Things (IoT) platforms for Human-to-Human (H2H), Human-to-Machine (H2M), Machine-to-Human (M2H), and Machine-to-Machine (M2M) communications. *T-Wing* makes it possible to host rich web content directly on their objects, which nearby objects can access instantaneously. Using a new mechanism that allows the Wi-Fi interface of the object to be turned on purely on-demand, *T-Wing* achieves very high energy efficiency. We have implemented *T-Wing* on an embedded board, and present evaluation results from our testbed. From the evaluation result of *T-Wing*, we compare our system against alternative approaches to implement this functionality using only the cellular or Wi-Fi (but not both), and show that in typical usage, *T-Wing* consumes less than 15× the energy and is faster by an order of magnitude.

## 1. Introduction

The proliferation of mobile platforms has led to an increase in the use of the mobile web-based interactions [1]. Broadly deployed mobile devices, including many commercial off-the-shelf (COTS) such as smartphones and IoT devices are generally equipped with two indispensable communication modules: Wi-Fi and a cellular network interface. Users are willing to pay for mobile data plans, and can hence browse websites from a mobile platform, wherever they are, just as they would from a general computer system. However, in contrast to a general computer system, the mobile platform is with the user most of the time. This has led to a new class of applications that allow a user to push content over the Internet from the convenience of his mobile platform. For example, users use their mobile platforms to upload photos on *Instagram* [2], update status on *Facebook* [3], and send tweets across the globe using *Twitter* [4]. The trend is to make it easier not only for users to push interesting content to the Internet, but also for smart objects to make interactions with mobile platforms of users [5,6].

**Figure 1:**
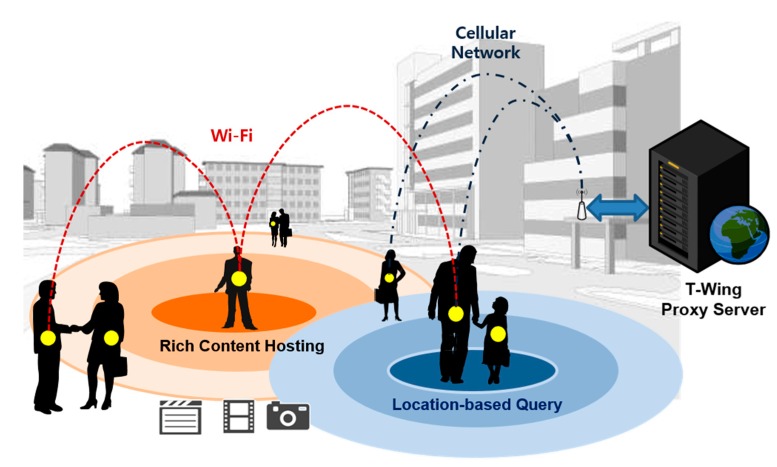
Overall concept diagram of Tiny-Web-Thing (*T-Wing*), a system that enables users to host and share content from their mobile devices.

In line with these trends, we propose Tiny-Web-Thing (*T-Wing*), a system that enables users to host and share content from their mobile devices. As shown in Figure 1, devices from all over the world can query for available mobile device webpages around a given location, and access them remotely. Furthermore, if devices are nearby, we enable them to share much richer content. Such a system enables several new applications. For example, when there is a happening or an event, users in the vicinity can share the occurrence worldwide within a short period of time. People can also use *T-Wing* to advertise themselves, which can be useful for financial transactions, or even for breaking the ice among strangers, for example in a dating scenario.

However, implementing *T-Wing* is non-trivial. For example, all content could be hosted over Wi-Fi to enable sharing of rich content. Nearby mobile platforms, which are in Wi-Fi range, can discover and access content over the Wi-Fi network at high throughput. However, such an approach is impractical since keeping the Wi-Fi interface on all the time quickly drains the mobile platform’s battery. This can be mitigated to some extent by turning the Wi-Fi interface on periodically, and broadcasting a beacon message. However, turning the Wi-Fi interface on and off may cause spikes in energy consumption [7]. Furthermore, in the absence of clever synchronization mechanisms among mobile platforms, such sleep and awake schedules would significantly increase the discovery time of two mobile platforms [8].

An alternative is for mobile platforms to transfer their content (along with their location) to a server on the Internet using the cellular network interface like Microblogs [9]. Although this approach is promising, it suffers from three problems when serving large content. First, the cellular uplink bandwidth is a severe constraint for mobile platforms in comparison to downlink bandwidth even in 4G (LTE) networks [10]. If several mobile platforms serve content using the same uplink connection, the experience when browsing mobile content will further degrade. Second, the battery power of mobile platforms is limited, and the cellular network interface causes significant energy drain when it is active [11,12]. It has been shown to have much lower bits/Joule than Wi-Fi. Although the actual power consumption may be changed in accordance with the usage of a specific network technology, the difference in energy efficiency among the heterogeneous network technologies makes this work more useful [13,14,15]. Finally, users of mobile platforms may pay for cellular data usage on a pay-as-you-go pricing model. Nowadays, despite the ever-increasing need of people for staying ‘connected’ at any time and everywhere, in many areas of the world data connection is extremely expensive [16]. Therefore, users will get charged for uploading their content, as well as downloading content from other object or people, thereby reducing the incentive to host content.

*T-Wing* achieves the desired properties not only in an energy and but also in a cost-efficient manner. In particular, it allows users to host content from their mobile platform. Data-intensive content can be downloaded directly from nearby mobile platforms using the high-bandwidth Wi-Fi interface. For instance, movies or other large files can be shared among nearby objects very efficiently, without requiring first uploading the data to the cloud via a cellular interface. Crucially, *T-Wing* is energy efficient. In normal operation, the mobile platform’s battery consumption is only minimally affected.

One critical component underlying *T-Wing* is a new mechanism that allows mobile platforms to turn on their Wi-Fi interface purely ‘on-demand’, that is, only when the mobile platform is either requesting data from nearby mobile platforms, or serving its content to any nearby mobile platform. The mechanism employs a proxy server in the cloud, which keeps track of each mobile platform’s location, and is able to predict for a given mobile platform, which other mobile platforms are within its Wi-Fi connectivity range. A mobile platform can then download data-intensive content directly from any such mobile platform: using its cellular interface, a mobile platform sends a request to the proxy server, which then asks both mobile platforms to turn on their Wi-Fi interface, and assigns non-conflicting IP addresses. The two mobile platforms can then connect directly in a peer-to-peer fashion and exchange data.

We have implemented *T-Wing* on an open hardware embedded board, called BeagleBoard [17] in which each mobile platform runs a local web server. The proxy server is implemented using Apache and SQL server. We compare our system against alternative approaches to implement this functionality using only the cellular or Wi-Fi (but not both), and show that in typical usage, *T-Wing* consumes less than 15× the energy and is faster by an order of magnitude.

The remainder of the paper is organized as follows: in Section 2, we review related works and analyze existing systems. In Section 3, we present the motivation of this work and our approach. In Section 4, we illustrate the core mechanism for on-demand Wi-Fi connection to nearby mobile platforms. In Section 5, we present the overall system design and components of the proposed system. In Section 6, we discuss the overall architecture of *T-Wing* and additional extensibility of this work. In Section 7, we evaluate the performance of the proposed system. Finally, in Section 8, we present our conclusions.

## 2. Related Work

In an attempt to meet the demands of an interactive mobile services for sharing of rich content, many studies have been performed and commercial services are launched. In this section, we present some of the related work in line with these trends.

Commercial products, such as *ShoZu* [18], make it easy to upload content from a mobile to social networking or content distribution sites, such as *Instagram* [2], *Facebook* [3], and *Youtube* [19]. However, they do not provide a mechanism to discover content shared by nearby mobile devices, or mobile devices at a specific location. *uLocate*’s service, called *WHERE* [20], allows users to geo-tag a photo uploaded to the web server. *Twitter* [4] and *Xumii* [21] enable mobile users to share content with their social network, just as they would communicate with them using their PC. However, they too do not have a concept of location of the tweeting phone. *Twitter* announced that tweets can be geo-tagged with the location of the phone. However, the details of this system are unknown.

Mobile Web Server (*MWS*) powered by Symbian [22] allows mobile phones to host a web server. The content of the web site can be accessed from a server on the Internet, which redirects the http requests to the mobile phone. The motivation of *MWS*, instead of hosting on the server, is that the server might be expensive when sharing only with a few people, for example, the family members. Similar to *MWS*, *T-Wing* enables mobile devices to host content. However, *T-Wing* is targeted to allow anyone to access the website while providing a richer experience to nearby mobile devices enhanced with ‘on-demand’ Wi-Fi connection to nearby mobile platforms.

From the energy efficiency perspective in mobile network, many studies have been performed. In [13,14], the authors compare the energy consumption of LTE, Wi-Fi and aggregate interface on a mobile device. Their results show that Wi-Fi consumes around 20% of LTE’s energy, and aggregate interface consumes 57% of LTE’s energy for a download of 8 MB of data. In [15], the authors study energy consumption of YouTube for mobile devices. The authors present a measurement study of YouTube’s energy consumption characteristics for network access technologies such as WCDMA and Wi-Fi. The results show that during a progressive download of content over Wi-Fi consumes around 40% of energy consumed by cellular WCDMA. Although the actual power consumption may be changed in accordance with the usage of a specific network technology, the difference in energy efficiency among the heterogeneous network technologies makes this work more useful [13,14,15].

In efforts to obtain behavioral patterns, many studies are being carried out for determining user context [23,24]. They attempted to recognize activities using a single three-axis accelerometer worn near the pelvic region and formulated the activity recognition as a classification problem. Google Play Services [25] provides special APIs for activity recognition, which returns the user activity. In this work, we incorporated the previous studies [23,24] into our system as an underlying context-aware mechanism.

*Micro-Blogs* [26] presented a social network framework for mobile devices to share blogs and content with phone users around a location. Information about the phone and their location is stored in a central server over the Internet. The server has a web front end over which users can query phones around a location. Sensing as a service (S^2^aaS) [27] is to provide sensing services using mobile phones via a cloud computing system. They identified unique challenges of designing and implementing the S^2^aaS cloud. However, this work mainly focusses on cloud computing model interacting with mobile devices to provide various sensing services using mobile devices for a large number of cloud users. SOR [28] presents a vehicular social network to enable social communications and interactions among users on the road during their highway travels. They address the fundamental privacy preservation issue to protect the social interest information of users during social communications. *meetMoi* [29] is a mobile dating tool. It stores a user’s profile on a server and constantly uploads the phone’s location. Users can then query the server to learn about nearby phones of users that are interested in dating. Mobile *Push-to-talk* [30] uses peer to peer connections for voice.

While our approach is similar to above previous works and commercial services from the end user’s point of view, this work presents a new mechanism that allows mobile platforms to turn on their Wi-Fi interface purely ‘on-demand’, that is, only when the mobile platform is either requesting data from nearby mobile platforms, or serving its content to any nearby mobile platform. We also look at the scalability of such an approach, when there are several hundred mobile devices, which can be moving as well. Our approach also enables users to share rich content with peers that are nearby. Further, our task has been to provide a full-fledged mobile platform tailored for a mobile computing environment, where numerous mobile devices and smart objects interact with each other. To accomplish this task, we thoroughly reviewed the ways by which the conventional mobile platforms and mobile network technologies are used in the environment and we consequently derived the blueprints for an efficient mobile platform, called *T-Wing*. Consequently, this work makes the following three contributions. First, we present a mechanism for mobile devices to host location-based content. Second, in a scenario when devices are nearby, we present a technique for accessing much richer content from the mobile devices. Third, we present new applications for mobile devices, which are triggered by *T-Wing*, i.e., the ability for mobile devices to host location based content on the Internet. We believe that users will develop several creative applications if provided with the mechanism of *T-Wing*.

## 3. Motivation

The goal of *T-Wing* is to allow users to host rich content from their mobile devices. Furthermore, this content should be instantaneously accessible to users that are nearby. In this section, we first outline the challenges in accomplishing this goal and then provide an overview of our approach.

### 3.1. Challenges

We describe the challenges in the design of *T-Wing* by outlining two simple solutions, and showing why they are not practical for hosting rich content from mobile devices.

- *Host over Wi-Fi*: A simple solution is to host all the content over Wi-Fi. Mobile devices can keep the Wi-Fi interface turned on all the time, and beacon information about themselves. Nearby devices, which are in Wi-Fi range, can discover and access content over the Wi-Fi network. The advantage of this approach is that the Wi-Fi interface supports high throughput and large files can be downloaded very quickly. However, keeping the Wi-Fi interface in the on state all the time will quickly drain the battery of the device, thereby significantly impacting the user experience.
- *Host over the Internet*: An alternative is for mobile devices to transfer their content to a server on the Internet. In most cases, since users will want their data to be available instantaneously, this content will be uploaded over the cellular data interface as this connection is mostly available. Mobile devices also upload their location to the server. A query from a mobile device would then return all the nearby mobile devices and their content would be delivered over the Internet. This approach is similar to the ones used by *Micro-Blogs* [26].

Although this approach is promising, it suffers from three problems when serving large content. First, cellular uplink bandwidth is a severe constraint for mobile devices. This bandwidth is not expected to increase beyond 50 Mbps in 4G (LTE) networks [31]. If several mobile devices serve content using the same uplink connection, the experience when browsing mobile content will suffer significantly. Second, the battery power of mobile devices is limited, and the cellular network interface causes significant energy drain when it is active [32,33]. It is because the communication over the Wi-Fi is more energy efficient than the communication over the cellular network from the energy efficiency (bits/Joules) perspective [11,12,13,14,15]. Finally, in several countries, the users still pay for cellular data usage on a per-packet basis. Therefore, users will get charged for uploading their content, as well as downloading content from other people, thereby reducing the incentive to host content.

Finally, in several countries, the users still pay for cellular data usage on a per-packet basis. Therefore, users will get charged for uploading their content, as well as downloading content from other people, thereby reducing the incentive to host content. Our study can be applicable into next generation mobile networks such as 5G since gaps between cellular network and Wi-Fi may still exist from the network performance perspective and from the energy efficiency (bits/Joule) perspective [11,12,13,14,15].

### 3.2. Overview of Our Approach

*T-Wing* solves the above problems by leveraging the Wi-Fi and cellular data interface in a cognitive manner. Users are provided with a simple interface to host web pages and content on their mobile device. Compressed versions of these web pages are uploaded to a central proxy server on the Internet. Mobile devices also upload their location to this server. When a user wants to query for nearby mobile devices, the request is handled by the proxy server, which responds with all mobile devices within a specified radius. It also uses an algorithm to predict mobile devices to which direct Wi-Fi connectivity is possible. If a user selects direct connectivity, the proxy server coordinates between mobile devices to quickly establish a Wi-Fi connection. Using this technique, mobile devices are able to download large content over Wi-Fi, while ensuring that the Wi-Fi interface is on only when it is needed.

## 4. On-Demand Wi-Fi Connection to Nearby Mobile Devices

A key component of *T-Wing* is a mechanism that allows mobile devices to communicate peer-to-peer over Wi-Fi to share large content. We note that several approaches in the research literature have proposed the use of peer-to-peer communication over Wi-Fi, but none of them have become popular because of Wi-Fi’s power consumption. This is because Power Save Mode (PSM) of IEEE 802.11 only works when the Wi-Fi card is associated to an Access Point (AP). This limitation will continue to hold with Wi-Fi direct. In *T-Wing*, we overcome this problem by enabling the Wi-Fi card only when it is required, and we accomplish this by intelligently using the cellular network interface on the mobile device. We describe the details in the rest of this section.

### 4.1. Discovery and On-Demand Wi-Fi Activation

In our mechanism, the Wi-Fi radio is activated purely ‘on-demand’, i.e., only when it is needed for accessing or serving content. To accomplish this on-demand activation of Wi-Fi, the mechanism uses a proxy server on the Internet. Every mobile device periodically uploads its location as well as its received signal strengths (RSSI) from neighboring cellular base stations to the proxy server over its cellular data (3G/4G LTE) connection. Since users keep their cellular data connection on most of the time, for applications such as push e-mail, this data can be sent to the server at little extra energy cost. We quantify this overhead in Section 7.

**Figure 2:**
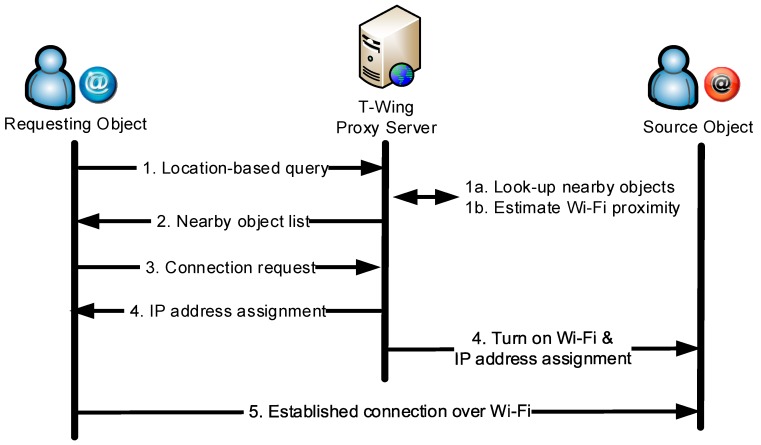
*T-Wing* protocol for discovery and on-demand Wi-Fi activation.

Figure 2 shows the *T-Wing* protocol for discovery and on-demand Wi-Fi activation. A mobile device can query for a list of nearby mobile devices that are hosting content. This query is first routed to the proxy server on the Internet. The server then uses an algorithm to predict if direct Wi-Fi connection is possible between the querying mobile platform and any nearby mobile device based on the RSSI values reported by the mobile devices. If a mobile device wants to access data from such a nearby mobile device, it activates its own Wi-Fi card, and the proxy server on the Internet notifies the mobile device hosting the content to turn on its Wi-Fi connection as well.

### 4.2. Establishing Connectivity

After the Wi-Fi radios are turned on, the next step is for mobile devices to connect to the appropriate BSSID (Basic Service Set Identifier), get an IP address and learn the IP address of the other mobile device. In our current implementation, mobile devices of *T-Wing* are equipped with N150 micro wireless adapter [34] as a Wi-Fi network module which works in IEEE 802.11n. The Wi-Fi network modules of *T-Wing* are configured for ad-hoc mode to communicate with nearby other *T-Wing* devices. All mobile devices participating in *T-Wing* are configured to use the same channel number. After the mobile devices get connected to the appropriate BSSID, they are assigned a non-conflicting IP address by the proxy server, which thus takes on the role of a kind of DHCP (Dynamic Host Configuration Protocol) server. We note that the mobile devices cannot use a real DHCP server, since this is a peer-to-peer network. Also, it would be highly inefficient to use autoconfig to assign themselves a non-conflicting link-local IP address since autoconfig takes 10 s of seconds to send an ARP request and avoid conflicts. Finally, we note that simply selecting IP addresses at random is insufficient with IPv4. IPv6 would be the perfect solution for this problem: it has a very large number 2^38^ of available link-local addresses. Specifically, due to the Birthday Paradox, there is a high likelihood of an IP address conflict even with relatively few neighbors in proximity. When the proxy server notifies the mobile device to wake up, the mobile device is assigned a link-local IP address. This address is only valid for the current network association between a mobile device requesting the content and a mobile device hosting the content, i.e., until the Wi-Fi connection is turned off. Similarly, the mobile device requesting the content is also assigned an IP address by the proxy server, and the proxy server informs the requestor of the IP address of the mobile device hosting the content. At this point, the mobile device can access the hosted content over IP from the nearby mobile devices.

Assuming that a mobile device participating in *T-Wing* makes a Wi-Fi connection just for a one-time accessing of small size of web content in a peer-to-peer manner, it may hinder user experience. This is because the network association consists of several operational steps. These include: (1) a connection to the appropriate BSSID; (2) an IP address assignment; and (3) a reception of the IP address of the other mobile device. However, once the network association is successfully completed, *T-Wing* does not require additional network configuration for a continuous web transaction with the mobile device hosting rich content; hence, *T-Wing* can provide an energy efficient communication channel with a relatively small percentage of network association overhead, in case that the mobile device accesses directly the mobile device hosting rich content over the Wi-Fi.

Finally, in case the Wi-Fi radio of a mobile device is already connected to an AP while being asked to connect by a neighboring mobile device, we can use Virtual Wi-Fi [35] to simultaneously connect to the Wi-Fi network as well as the *T-Wing* peer-to-peer network. This solution has the extra advantage that it seamlessly scales to the setting when a client wants to access content from multiple mobile devices.

### 4.3. Predicting Wi-Fi Connectivity between Two Mobile Devices

One important building block of the above mechanism is the algorithm to predict whether two mobile devices are within Wi-Fi range. As discussed, we cannot find the natural way of determining Wi-Fi connectivity, i.e., by using Wi-Fi beacons, or by using probe requests/responses, because during normal operation the mobile devices’ Wi-Fi interface is turned off. Although there are several energy impact which affects the battery life time like security mechanisms for the mobile devices [36,37] in a realistic scenario, periodically turning it on and off would result in a prohibitive battery drain. Furthermore, we want to maximize the accuracy of the *T-Wing* proxy server’s prediction about which mobile devices are within Wi-Fi range of a requestor’s location. For this purpose, using standard, simple localization techniques based on 3G/4G LTE (e.g., based on cell tower that you are located in, or even sophisticated localization techniques based on received signal power strengths and triangulations) is not sufficiently accurate to predict Wi-Fi connectivity between two mobile devices.

Our solution is based on the observation that we do not need to determine the exact ‘location’ of any mobile device, but instead we only need an estimate for the ‘relative RF distance’ between the mobile devices. In other words, we are merely interested in ‘the similarity of locations of two mobile devices locations’. As it turns out, this is a structurally easier problem, and it allows us to achieve much more accurate results than those based on estimating the proximity of the given two nodes’ measurements.

We have implemented and evaluated two algorithms for Wi-Fi connectivity prediction. When predicting the existence of Wi-Fi connectivity between two mobile devices *A* and *B*, both algorithms compare the received signal strengths from base stations as measured at *A* and *B*, but they differ in the exact prediction metric. In the first metric (Metric *A*), each mobile device reports its *X* strongest base stations, and the algorithm computes the pair-wise differences of RSSI values on the two mobile devices of these *X* strongest base stations. We have tested different values for *X*. In the second metric (Metric *B*), each mobile device reports RSSI values from all base stations whose RSSI exceeds −15 dBm. The algorithm then computes for each such base station the difference between the two RSSI values and takes the maximum or average of these values to predict connectivity. The above metrics are still work in progress and we are investigating other approaches as well.

### 4.4. Location-Update Adaptation Algorithm

**Figure 3:**
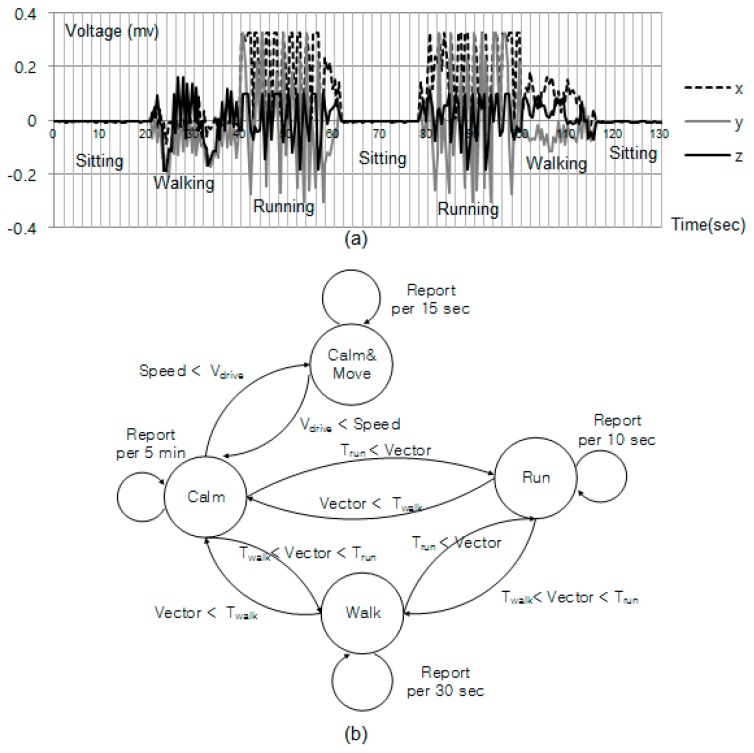
(**a**) The raw accelerometer readings along the three axes for sitting, walking, and running, (**b**) State diagram for duty-cycle scheduling.

Our mechanism requires mobile devices to periodically update the proxy server with their location. In efforts to obtain behavioral patterns, many studies are being carried out in exploiting this property for determining user context [23,24]. They attempted to recognize activities using a single three-axis accelerometer worn near the pelvic region and formulated the activity recognition as a classification problem. In this study, we seek to minimize use of the localization module, and avoid unnecessary transmissions over the data network for energy-efficiency. Our approach is based on the observation that a mobile device is stationary most of the time, and if we only update the location when it changes significantly, the impact on battery life will not be significant. To detect when the mobile device has moved, we use the well-known approach of reading the accelerometer sensor on the mobile device. Data from the accelerometer sensor has the following attributes: time, acceleration along *x* axis, acceleration along *y* axis, and acceleration along *z* axis. At a sampling frequency of 25 Hz, the raw accelerometer data is stored on a windows size of 250 in a first-in-first-out manner. Therefore, the window represents data for previous 10 s. The window of several seconds can sufficiently capture cycles in activities such as walking, running. In this work, the mean (absolute value of average) of acceleration (*x*-, *y*-, and *z*-axes) was used to recognize user’s activities because it is one of the most accessible measures of time series data [23]. We set the parameters V_drive_, T_run_, T_walk_ to 20 miles/h, 0.12 mv, 0.06 mv, respectively. The parameters V_drive_ is configured for the mobile devices equipped with GPS module. We note that the parameter values T_run_, T_walk_, may vary depending on which a three-axis accelerometer is used in the mobile device. Figure 3a shows the raw accelerometer readings along the three axes for sitting, walking, and running for one person carrying the mobile device. As expected, the sitting traces is flatter than when the person is walking and running. The peaks in the walking and running traces are a good indicator of footstep frequency. When the person runs a larger number of peaks per second is registered than when people walk. The usefulness of these features has been demonstrated in prior work [23,24]. Given these observations, we find that the mean, standard deviation, and the number of peaks per unit time are accurate feature vector components, providing high classification accuracy [23]. Given these observations, we find that the mean, standard deviation, and the number of peaks per unit time are accurate feature vector components, providing high classification accuracy [23]. Based on these readings we devise an algorithm to determine when the location update should be sent to the proxy server as described in Figure 3b.

## 5. Design of *T-Wing*

The mechanism described in Section 4 provides the key functionality on top of which we have designed *T-Wing*. Specifically, each *T-Wing* object is represented by a unique ID. The ID is created by the user when setting up a *T-Wing* account, and is used by the proxy server when returning the list of nearby mobile devices. Specifically, when a mobile device queries for nearby mobile devices, the proxy server responds with the friendly names of nearby mobile devices. A user can maintain a profile as well as miniaturized form of a webpage, which contains a small set of information, but no data-intensive content. Profiles and mini-webpages are also stored on the proxy server and returned along with the list of nearby mobile devices.

The *T-Wing* frontend on the client then depicts the list of nearby mobile devices on the screen. It is also possible for users to use filters such that only users with certain characteristics in the profile are depicted, and users can also specifically search for certain profiles among the users within their neighborhood. Finally, an icon is depicted next to the friendly name of any mobile device that the algorithm predicts to be within Wi-Fi range: This indicates that the user can access all content directly from this nearby mobile device in case of assuming the prediction is correct. The requestor’s mobile device turns on the Wi-Fi radio, and the proxy server on the Internet notifies the mobile device hosting the content to turn on its Wi-Fi connection as well. In the unlikely case that the prediction was false, the user will be informed that no Wi-Fi connectivity could be established.

### 5.1. Hosting Content

We enable mobile devices to host a web page that can be accessed by other mobile devices over the http port. All content that a user wants to share, e.g., pictures, videos, etc., is linked via its main web page. For *T-Wing*, we have designed our own stripped-down version of a Web server, which we call *T-Wing-Provider*. It is capable of handling multiple incoming requests and serves various media formats, including html, pictures and video. We describe the details of our *T-Wing-Provider* implementation in Section 6.

### 5.2. Miniaturizing a Mobile Platform’s Content

*T-Wing* uploads a compressed version of web pages to the central proxy server on the Internet. Savings in bandwidth are possible by considering the information redundancy. Traditionally, this has been done by applying widely used lossless compression such as Gzip [38], Bzip [39]. Instead, we take an adaptive compression approach [40,41] and show that the size of content can be significantly improved. By considering the entropy characteristics of web pages, we were able to apply size reduction techniques as shown in Table 1. We first use lossy compression to discard as many bits as possible for image, audio, and video data and then we applied lossless compression for the remaining text data with LZO compression [42] which has a fast compression and decompression time.

### 5.3. Handling Multiple Clients

If several users want to simultaneously access the same content from a mobile device, several optimizations are possible. One is the concept of *content proxying*, which allows a popular content provider mobile device to increase its upload fan-out by replying to a new requestor with a list of other mobile devices that have previously downloaded the same content. In case any of these mobile devices is still in Wi-Fi range of the new requestor, it may be faster for the new requestor to download the content from there. Further optimizations include smart IP-address management by the proxy server, as well as changes to the Wi-Fi scheduling policy to avoid the rate anomaly problem.

- *IP-Address Management*: As mentioned above, the proxy server assigns IP addresses to mobile devices upon request. If a second requestor device wants to access content from a mobile device that is already serving content, then the proxy server must not, of course, assign a new IP address to this device. Instead, this server device’s IP address is simply conveyed to the requestor, who can then access this device by connecting via Wi-Fi. In order to be able to assign the same IP address to a subsequent requestor, the server thus needs to keep track of the IP addresses that are currently in use, and more generally, it needs to know which device has its Wi-Fi in use, and which device does not. For this purpose, we use a concept of ‘IP-address leases’: a device, after having been assigned an IP address, needs to report to the proxy server whether it is still actively using its Wi-Fi radio. If the device does not renew its lease on time, the server knows that the Wi-Fi is no longer in use and returns the IP address to the pool.
- *Content Proxying*: If many mobile devices attempt to access the same content from the same device, this device may be unable to serve all requestors efficiently due to lack of bandwidth. In *T-Wing*, we use the concept of *content proxying* to alleviate this problem to some degree. Assume that three mobile devices *A*, *B*, and *C* access the same content from device *S*, but that *A* was the first to successfully complete the download because its request was issued before the two others. Now, when *B* and *C* request the same files, the device *S* can serve one of the requests itself, say request *B*, but it can also tell device *C* that *C* might be able to download the file faster from content proxy *A*. If device *C* is within Wi-Fi reach of device *A*, *C* can then request the file from *A*, rather than requesting it from the original device *S* directly, which enables the transfers from *S* to *B*, and from *A* to *C* to go in parallel, rather than having *S* serve both requestors *B* and *C* sequentially. In other words, *T-Wing* uses the concept of content proxying to opportunistically increase the fan-out of the serving device, and thus increase the overall distribution speed.

## 6. Architecture of *T-Wing*

We have implemented *T-Wing* as a combination of an application of mobile platform and a service in the cloud. We explain both of these in more detail in the rest of this section.

**Figure 4:**
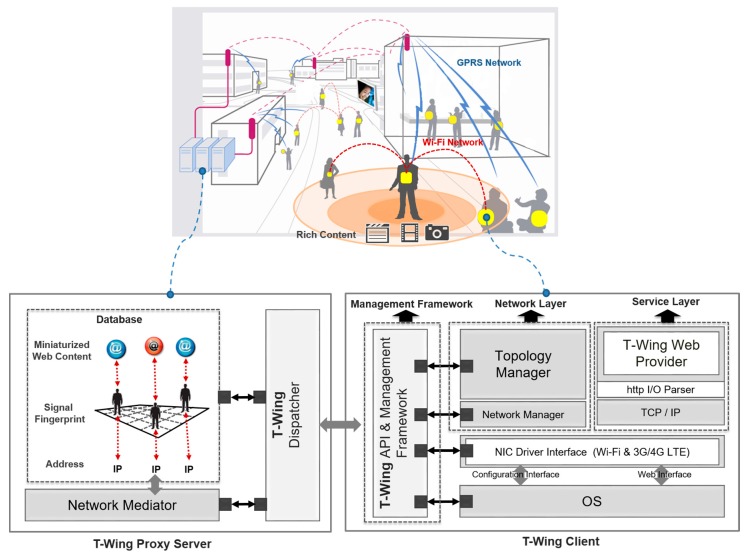
Overall architecture of *T-Wing* that consists of *T-Wing* proxy server and *T-Wing* client.

**Figure 5:**
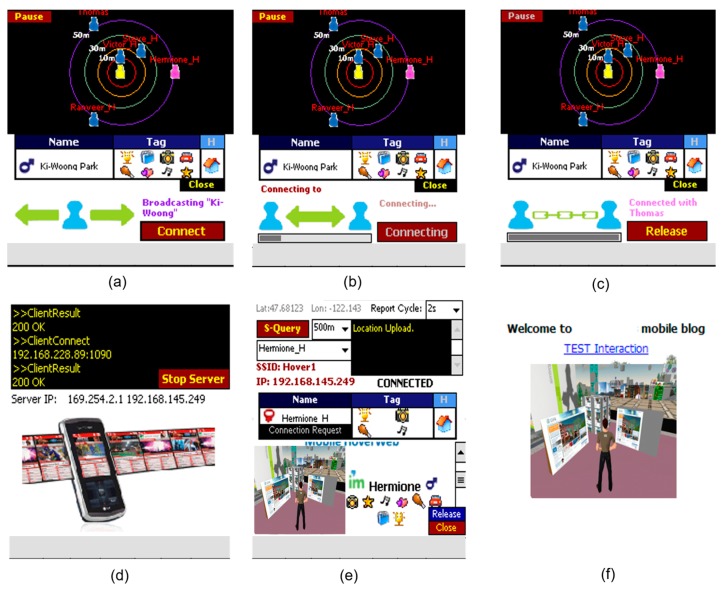
Screenshots of *T-Wing* on an embedded platform: (**a**) Searching nearby users; (**b**) Connecting over Wi-Fi network interface; (**c**) Established connection over Wi-Fi in a peer-to-peer manner; (**d**) *T-Wing* web provider with log messages; (**e**) Miniaturized web page; (**f**) Web browsing with rich content.

- *T-Wing Client*: The architecture of a *T-Wing* client is illustrated in Figure 4. It has three main components: the service, management and network layers. The service layer implements the web server functionality, the network layer establishes the connection between nearby mobile platforms, and the management layer coordinates interactions between the two and also communicates with the *T-Wing* proxy server. For the service layer, we implemented our own version of a web server on the mobile platform. Since the processes of the web server on the mobile platform tend to consume a lot of memory as the number of parallel connections grows, it’s important to keep memory usage down. The web server on the mobile platform limited the maximum number of parallel connections to three for keeping a small memory footprint. The maximum number of parallel connections can be modified in the environment setting of *T-Wing*. The network layer provides a number of wrapping functions to set the IP address, IP routing table, and to enable/disable NIC. The management component reports the mobile platform’s location to the proxy server, and also communicates with it when the user requests to see nearby *T-Wing* users. Also, if a nearby mobile platform is interested in accessing content hosted on the mobile platform, the proxy server contacts the management framework to enable Wi-Fi and set up a network connection.
- *T-Wing Proxy Server*: The proxy server consists of a database and a web service. The database maintains the miniaturized web page for every object, its location and cellular signal strengths from base stations. The web service takes requests from clients, determines nearby mobile platforms and coordinates connectivity among the mobile platforms as described in the previous section. *T-Wing* proxy server is an indispensable component, which means that *T-Wing* clients cannot be operated in case that there is no coverage of cellular network. The main advantage of depending on the *T-Wing* proxy server is that users allow to host content from their mobile platform not only in an energy and but also in a cost-efficient manner. And data-intensive content can be downloaded directly from nearby mobile platforms using the high-bandwidth Wi-Fi interface.
- *T-Wing Screenshots*: Figure 5 shows screenshots of *T-Wing* on an embedded platform, BeagleBoard-xm. Figure 5a–c show screenshots which enable mobile devices to communicate peer-to-peer over Wi-Fi to share large content. Figure 5d presents a screenshot of a *T-Wing* web provider with log messages.
- *Nearby Searching*: It allows users to search the available *T-Wing* objects near them. It uses signal fingerprint to get the nearby object’s information and shows the surrounding *T-Wing* objects. When a user has chosen the *T-Wing* object the user wants, the user can make a direct connection to the *T-Wing* host by coordinating with the *T-Wing* Proxy Server.
- *T-Wing Web Browsing*: *T-Wing* provides the user with two kind of connection options, enabling the user to see the miniaturized web page over cellular network or enabling the user to see the entire web page over direct Wi-Fi connection as shown in Figure 5e,f, respectively.

## 7. Evaluation of *T-Wing*

In this section, we present the evaluation results obtained with our prototype version of *T-Wing*. We first present micro benchmarks quantifying the performance benefits from individual components that comprise *T-Wing*, and then show the overall performance of *T-Wing* and compare it to alternatives. We first quantify the uplink bandwidth needed to run *T-Wing*, energy consumed to maintain up-to-date location information in the cloud, and the accuracy of our Wi-Fi connectivity prediction algorithm. These evaluations are to highlight how the *T-Wing* improves the effective bandwidth when the compression module is properly applied and to quantify the energy savings achieved by the adaptive location-reporting algorithm, respectively.

### 7.1. Uplink Bandwidth Requirement

**Figure 6:**
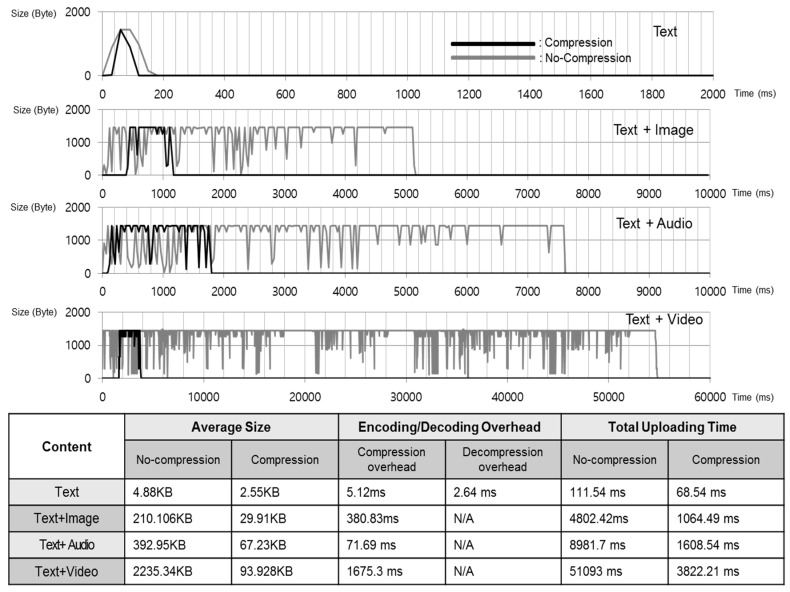
Time-series and measurements showing total uploading time through the cellular network.

Figure 6 shows the time to upload different file types through the cellular data network. In order to measure the time to upload different file types through the cellular data network, we performed this experiment on an embedded platform, Beagleboard-xm [17] equipped with an AM37× 1 GHz (ARM Cortex-A8 compatible) processor, a Wi-Fi and a 3G/4G LTE dongle (Huawei^TM^ E3276) [43] which is connected over the 3G network system though it supports GSM/GPRS/EDGE communication modes. We measured the total time to upload different file types from a mobile device participating in *T-Wing* to the *T-Wing* proxy server through the cellular data network.

In our experiment, we transferred our benchmark files through the cellular data network for each scenario. We then measured the improvement in transfer performance enabled by the compression strategy described in Table 1. Since the cellular uplink bandwidth is a severe constraint for mobile platforms, the total uploading time is much faster compared to the original file even when compression/decompression overhead is included. On average, we found that performance is improved by 4× for the content we measured.

### 7.2. Energy Overhead of Reporting Location

**Figure 7:**
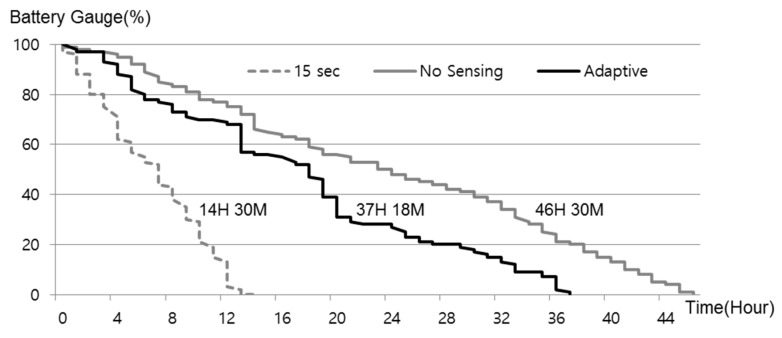
Battery life time with varying location update duty cycle.

To quantify the energy savings achieved by the adaptive location-reporting algorithm described in Section 4, we first measured the battery life time of the embedded platform described in Section 7.1. We fully charged the battery (2850 mAh) of the mobile platform and then measured the battery life time for each case (report per 15 s, adaptive report, no report) as described in Figure 3a. We assumed that 90 percent of personal life cycle is in the “Calm State”, while the remainder is in “Run State” or “Walk State”, 2 and 8 percent, respectively [44]. For the measurements of the energy saving effect by the adaptive location-reporting algorithm, the battery life time was measured with turning the Wi-Fi interface and process of web provider off. As shown in Figure 7, our results show that using our adaptive location update protocol, users can greatly increase the total usage lifetime of *T-Wing*.

### 7.3. Accuracy of Wi-Fi Connectivity Prediction

**Figure 8:**
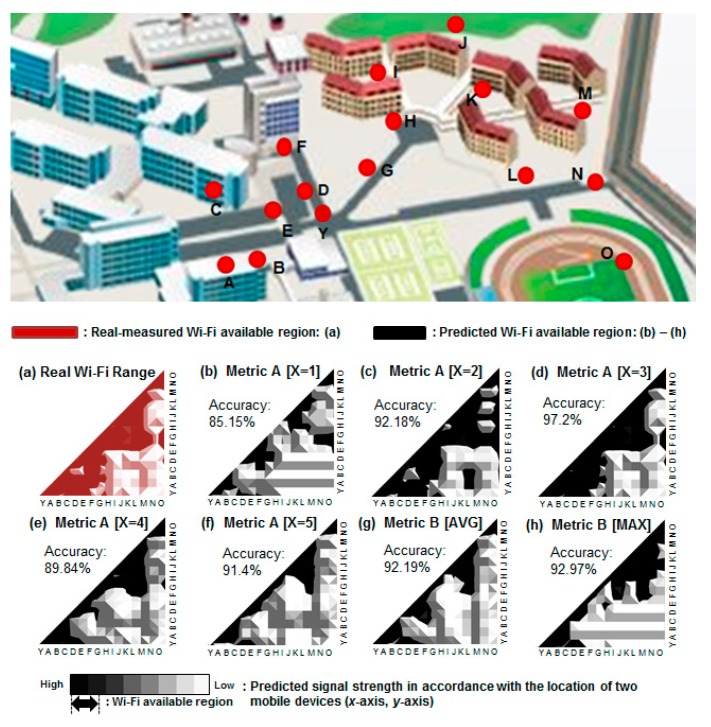
Performance of the Wi-Fi connectivity prediction algorithm for different metrics and values of ‘X’.

We evaluate the accuracy of our Wi-Fi connectivity prediction algorithm as described in Section 4.3. We measure the prediction accuracy at 16 different locations. Figure 8 shows the accuracy for different values of *X* for Metric *A* (i.e., the number of base stations reported to the proxy server) as well as Metric *B*. The best results are achieved for Metric *A* with X = 3 when the accuracy reaches 97.2%. We verified that this result is consistent across different regions and at different time.

### 7.4. Performance Evaluation

We compare the performance of *T-Wing* against two alternative implementations, one that only uses Wi-Fi network and other that only uses cellular network. We contrast our approach using two metrics: energy and response time.

**Figure 9:**
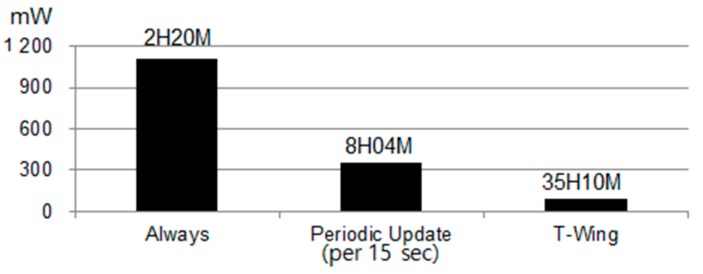
Energy consumption with varying communication mode.

**Figure 10:**
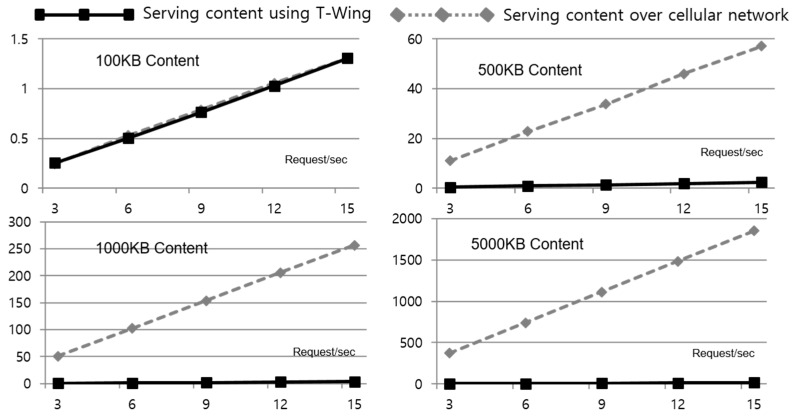
Response time when serving content using *T-Wing* compared to downloading over the cellular network.

- *Energy Consumption*: To quantify the energy consumption of *T-Wing*, we measured the power consumption of the embedded platform. For this measurement, we assumed that 10 web page requests arrived per hour, and every user sends 10 location-based query and web page requests per hour. We assumed the size of every web page is 500 KB. Table 2 illustrates the power consumption of each state. The mobile platform expends severe battery power to keep its Wi-Fi interface on when it is broadcasting and communicating. Even when the Wi-Fi radio is connected, the device consumes more than about 10 times the battery power than in cellular network interface on. These numbers indicate that the total lifetime of a mobile platform can be significantly increased if the Wi-Fi radio is turned off most of the time. Figure 9 plots the total communication energy consumption for one hour and the battery life time for the various communication mode. It shows the energy consumed in the wireless interface (Wi-Fi and Cellular network), compared to the energy consumption when utilizing *T-Wing*. Our base comparison is using the Wi-Fi in always on mode for the mobile platform. We save up to 95% of the energy consumption compared to always Wi-Fi or the periodic Wi-Fi mode.
- *Response Time*: We compare the response time of *T-Wing* when serving requests compared to an alternative implementation in which the web content is hosted in the cloud, and downloaded using the cellular network. Figure 10 illustrates the response time when varying the number of concurrent requests per second. There is not much difference when the content is small. However, the time to download content over cellular networks is significantly more for larger sized content.

## 8. Conclusions

In this paper, we present *T-Wing*, a tiny networked mobile platform which allows objects to host content from their mobile platform. Data-intensive content can be downloaded directly from nearby mobile platforms using the high-bandwidth Wi-Fi interface. One critical component underlying *T-Wing* is a new mechanism that allows mobile platforms to turn on their Wi-Fi interface purely ‘on-demand’, that is, only when the mobile platform is either requesting data from nearby mobile platforms, or serving its content to any nearby mobile platform. We have implemented *T-Wing* on an open hardware embedded board, BeagleBoard-xm in which each mobile platform runs a local web server.

## Figures and Tables

**Table 1:** Compression strategy for each content type.

Content Type	Compression Strategy
Text	Compression by LZO
Image	Trans-encode image to 640 × 280
Audio	Full audio file → cutting up first 5 s
Video	Full video file → cutting up first 5 s → trans-encode from .wmv to animated .gif

**Table 2:** Power consumption of the BeagleBoard for different states of its network interfaces.

Cellular	Wi-Fi	Power Consumption
Off	Off	23.17 mW
On	Off	31.82 mW
On (Send/Recv)	Off	358.27 mW
On	On (Broadcast)	1107.84 mW
On	On (Connected)	398.27 mW
On	On (Send/Recv)	1319.29 mW
